# Organ‐specific equivalent uniform dose constraints and radiobiological parameters for radiation treatment planning of abdominal tumors

**DOI:** 10.1002/acm2.70401

**Published:** 2025-12-28

**Authors:** Samira Dabaghmanesh, X. Allen Li, Eric S. Paulson, Beth Erickson, William Hall, An Tai

**Affiliations:** ^1^ Department of Radiation Oncology Medical College of Wisconsin Milwaukee Wisconsin USA

**Keywords:** equivalent uniform dose

## Abstract

**Background:**

Radiation therapy planning (RTP) for abdominal tumors often requires multiple dose‐volume constraints for each organ at risk (OAR), which vary with different fractionation schemes. This variability can complicate biologically optimized treatment planning.

**Purpose:**

This study aims to extract fractionation‐independent dose constraints based on the concept of equivalent uniform dose (EUD), utilizing published clinical data for a range of fractionation regimens. The goal is to derive organ‐specific radiobiological parameters as well as the EUD for each OAR that can be used in biologically optimized treatment planning for abdominal tumors, independent of the specific fractionation scheme applied.

**Methods:**

Clinical dose‐volume constraints for duodenum, stomach, and small bowel were compiled from available literature sources. These dose constraints were obtained for conventionally fractionated radiotherapy (CFRT), hypofractionated RT, and stereotactic body radiotherapy (SBRT) and were associated with less than a 10% risk of grade 3 toxicity as categorized by CTCAE v.3 or v.4, RTOG, and EORTC. For each OAR, an iso‐EUD fitting with EUD calculated based on the linear‐quadratic (LQ) model or linear‐quadratic‐linear (LQ‐L) model and a dose volume histogram generated from these dose volume constraints for each fractionation was applied to extract model parameters such as *α*/*β* ratio and ‘*n*’ (the volume effect of the OAR). Based on the obtained parameters, the dose constraint in EUD and the equivalent physical dose in 2Gy fraction (EQD2) were calculated.

**Results:**

The EUD constraints for LQ‐L (LQ) models are 52.62 (55.10) Gy, 48.22 (48.60) Gy, and 46.10 (45.06) Gy, *α*/*β* values are 5.42 (5.59) Gy, 7.67 (6.76) Gy, and 12.15 (8.20) Gy, and ‘*n*’ values are 0.06 (0.01), 0.03 (0.02), and 0.06 (0.08) for duodenum, small bowel, and stomach, respectively. Additional two parameters for the three OARs in LQ‐L are 5.15 Gy, 8.65 Gy, and 5.20 Gy for *dt* and 5.40, 4.72, and 3.33 for *γ/α*. The LQ‐L model fits the clinical data better than the LQ model.

**Conclusions:**

The obtained *α*/*β* values are comparable with those published previously. The extracted EUD constraints together with the volume effect parameter ‘*n*’ can be used for plan optimization and evaluation.

## 1 INTRODUCTION

Radiation therapy (RT) plays a crucial role in cancer treatment by delivering effective tumor doses while minimizing damage to healthy tissues where possible. However, radiation inevitably affects normal tissues, potentially leading to organ toxicities. To optimize treatment outcomes and patient well‐being, radiation oncologists aim to predict and reduce side effects by refining treatment planning.

Radiobiological models, such as the linear quadratic model (LQ)^1^ and the linear‐quadratic‐linear model (LQ‐L)^,^ ^2^ are widely used to predict radiation effects. Conventionally, these models are applied using nominal radiobiological parameters (such as *α*/*β* ratio of 3 and 10 for late and acute responding tissues), but different tissues respond differently to radiation due to varying intrinsic radiosensitivity.^3^, ^4^, ^5^, ^6^, ^7^ Hence, a more precise method is needed to determine organ‐specific radiobiological parameters and calculate the biologically effective doses to normal tissues thus enables one to estimate the extent of toxicities. Using clinical data for this purpose is a viable approach which helps to improve treatment planning and plan evaluation.

Quashie et al.^8^ developed a feasible procedure to acquire organ‐specific radiobiological parameters for radiation therapy planning of head and neck organs. They employed LQ and LQL models to fit Biologically Effective Dose (BED) against clinical data in their investigation. However, their approach primarily focused on Maximum Point Doses (MPD) for extracting the model parameters, neglecting volume effects, thereby limiting its applicability to more serial organs. For the organs in the abdomen such as the duodenum, stomach, and small bowel, volume effects become significant, necessitating consideration of multiple dose‐volume constraints for treatment planning.

The equivalent uniform dose (EUD) is a valuable concept that incorporates the volume effect and is useful for evaluating the radiobiological impact of non‐uniform dose distributions. Initially proposed by Niemierko in 1997^9^ for tumors and subsequently extended to normal tissues,^10^ EUD represents the uniform dose that, if delivered over the same number of fractions as the non‐uniform dose distribution of interest, results in an equivalent radiobiological effect. The versatility of EUD lies not only in its predictive power for toxicity^11^, ^12^, ^13^ but also in its ability to simplify complex dose plans into a single metric, providing a more comprehensive description of the biological effect of a non‐uniform dose distribution. It also found its way in wide application in various aspects of radiation therapy, including plan optimization (as a valuable endpoint for evaluating and comparing treatment plans) featuring different fractionation regimens and modalities,^14^, ^15^ serving as an optimization parameter in inverse planning systems, guiding adaptive radiotherapy, and re‐irradiation protocols.

In this study, we apply the EUD formulation to extract organ‐specific radiobiological parameters from clinical data and obtain EUD and EQD2 for three abdomen organs, duodenum, stomach, and small bowel. These parameters can be used for biologically optimized treatment planning, particularly for estimating organ maximum dose constraints of different fractionations in RT planning for abdominal organs.

It is important to note that Quashie et al.’s analysis was limited to maximum point dose data and did not incorporate the volume effect or equivalent uniform dose (EUD) calculations. In contrast, our study extends this approach to abdominal organs, where dose–volume relationships are critical, by employing iso‐EUD fitting to obtain both radiobiological parameters and organ‐specific EUD constraints. There is no overlap between the current study and that of Quashie et al., as the scope, methodology, and organ sites investigated are distinct. Our work provides a more comprehensive framework for biologically optimized treatment planning of abdominal tumors.

## 2 MATERIALS AND METHODS

### 2.1 Study design

Clinical dose‐volume constraints corresponding to smaller than 10% of grade 3 toxicity were collected for three abdominal OARs—the duodenum, stomach, and small bowel across various RT fractionation schemes, including conventional fractionation RT (CFRT), hypofractionation RT (HFRT) and SBRT. Using the EUD approach together with the (LQ) and (LQ‐L) models, we would like to extract radiobiological parameters for each of the three OARs.

### 2.2 Data collection and toxicity criteria

We compiled and analyzed clinical dose‐volume constraint data for OARs from various literature sources, including the American Association of Physicists in Medicine (AAPM) Task Group (TG101),^16^ Hypofractionated Treatment Effects in the Clinic^,^ ^17^ the Quantitative Analysis of Normal Tissue Effects in the Clinic (QUANTEC),^18^ Timmerman constraints,^19^ and other national and international published studies (20, 21, 22, 23, 24, 25, 26, 27, 28, 29, 30, 31, 32, 33, 34 and also see the additional references that are listed in the supplement (Tables 1s–3s)).

The data, covering CFRT, HFRT and SBRT were analyzed to determine the physical MPD and dose‐volume constraints with a particular endpoint corresponding to no more than 10% grade ≥3 toxicity. The endpoints were selected according to toxicity scales categorized mostly by Common Terminology Criteria for Adverse Events (CTCAE v3 or v4)^35^ (Cancer Therapy Evaluation Program 2009) and the Radiation Therapy Oncology Group (RTOG)/European Organization for Research and Treatment of Cancer (EORTC).^36^ Specific endpoints included ulceration for the duodenum, ulceration/fistula for the stomach, and enteritis/obstruction for the small bowel, as per Timmerman's endpoints. The dose‐volume constraints gathered in this study pertain to single‐course RT, excluding reirradiation. The maximum dose constraints are represented either as the minimum dose to the highest 0.035cc OAR volume (D_0.035cc) or as MPD. Additionally, constraints associated with various volumes (e.g., D1cc, D2cc, D75cc) were also collected.

Tables 1s–3s in the supplementary material provide a summary of published clinical data for the three OARs across different modalities, including CFRT, HFRT, and SBRT. The tables also present the number of fractions, doses corresponding to different volumes, the use of chemotherapy or surgery, and their respective references.

### 2.3 Equivalent uniform dose

The EUD formulation as proposed by Niemierko Invalid source specified is given by:

(1) $$EUD={\left[\sum_{i=1}^{X}\left(v_{i}{D_{i}}^{a}\right)\right]}^{1/a}$$

here *X* is the total number of dose bins. $D_{i}$ denotes the dose received by a normalized sub‐volume $v_{i}$, and ‘*a*’ is a tissue‐dependent parameter related to the Lyman model parameter ‘*n*’ by *a* = 1/*n* (see Ref. 11). The parameter ‘*a*’ governs the organ's response to radiation, with positive values indicating normal tissues and negative values indicating tumors. A completely parallel tissue corresponds to *a* = 1, while a completely serial tissue approaches positive infinity. When *a* = 1, the EUD represents the mean dose over the entire volume.

### 2.4 Incorporating the fractionation

To accommodate different fractionation schemes, we use the concept of the equivalent physical dose in 2 Gy fraction (EQD2) (Equation 2). By integrating EUD with EQD2, we retain the advantages of EUD while accounting for fractionation effects and linear and quadratic survival characteristics.

(2) $$\begin{array}{c} EQD2=\frac{Nd\left(1+\frac{d}{\alpha /\beta }\right)}{1+\frac{2}{\alpha /\beta }}\;\;\;\;\;\;\;\;\;\;\;\;\;\;\;\;\;\;\;\;\;\;\;\;\;\;\;\;\;\;\;\;\;\;\;\;\;\;\;\;d\leq d_{t}\\ EQD2=\frac{Nd_{t}\left(1+\frac{d_{t}}{\alpha /\beta }\right)+\frac{\gamma }{\alpha }N\left(d-d_{t}\right)}{1+2/\alpha /\beta }\;\;\;\;\;\;\;\;\;\;\;\;\;\;\;\;\;\;\;\;\;\;\;d>d_{t} \end{array}$$

where N is the number of fractions and *d* is the fractional dose. α/β quantifies the sensitivity of a tissue to the change of fractionation in the LQ model, 𝛾 is the linear coefficient for the final linear portion of the survival curve, and $d_{t}$ is the transition dose at which LQ‐L behavior begins. The LQ model is a simplified version of the LQ‐L model assuming the linear‐quadratic term works regardless of the magnitude of fractional dose (d). Combining Equations (1) and (2) we derived the modified equivalent uniform dose mEUD^13^

(3) $$mEUD={\left(\sum_{i}\nu_{i}EQD2_{i}^{a}\right)}^{1/a}$$

The dose matrix will be converted to EQD2 on a voxel‐by‐voxel basis first and then the sum extends over volumes to obtain the mEUD.^37^ The mEUD concept is employed in different studies as a predictor of toxicity.^13^, ^37^, ^38^

Calculated from the differential dose‐volume histogram (dDVH), mEUD accounts for dose inhomogeneity and fractionation difference. The summation in Equation (3) runs from the lowest dose volume constraint in the data set for each OAR to the maximum dose constraint. The sub‐volume $v_{i}$ was normalized to the largest volume of the data set. Linear interpolation was used to link the two adjacent data points and form a truncated dDVH, which represents the volume of an organ that receives the highest doses and thus is associated with the radiation toxicity of the organ.

By fitting mEUD against clinical dose‐volume constraints, we extracted EUD, EQD2, α/β, γ/α and $d_{t}$, and n parameters of three abdominal OARs in which EUD and EQD2 correspond to the dose constraints with <10% of grade 3 toxicity.

Through the fitting process, non‐linear least‐squares minimization and Levenberg‐Marquardt curve fitting algorithms in MATLAB minimize the Chi‐square error, given by:

$$c^{2}=\sum_{N=1}^{N}{\left(EUD_{mean}-EUD\left(N\right)\right)}^{2}$$

where $EUD_{mean}$ (= mean (EUD(N)) represents the mean EUD over all fractions and EUD(N) is the EUD in each fraction. For each optimized value for the model parameters, we reported the 95% confidence intervals (CI). Additionally, we calculated the reduced Chi square, given by $r\_\chi^{2}=\chi^{2}/n\mathrm{df}$, where the number of degrees of freedom (ndf) equals the number of observations minus the number of fitted parameters. The number of fitted parameters are 2 and 4 for LQ and LQ‐L, respectively.

## 3 RESULTS

Based on the available clinical data, LQ‐L and LQ model parameters as well as EQD2 and EUD values for the three OARs were extracted and described below.

### 3.1 Duodenum

**FIGURE 1:**
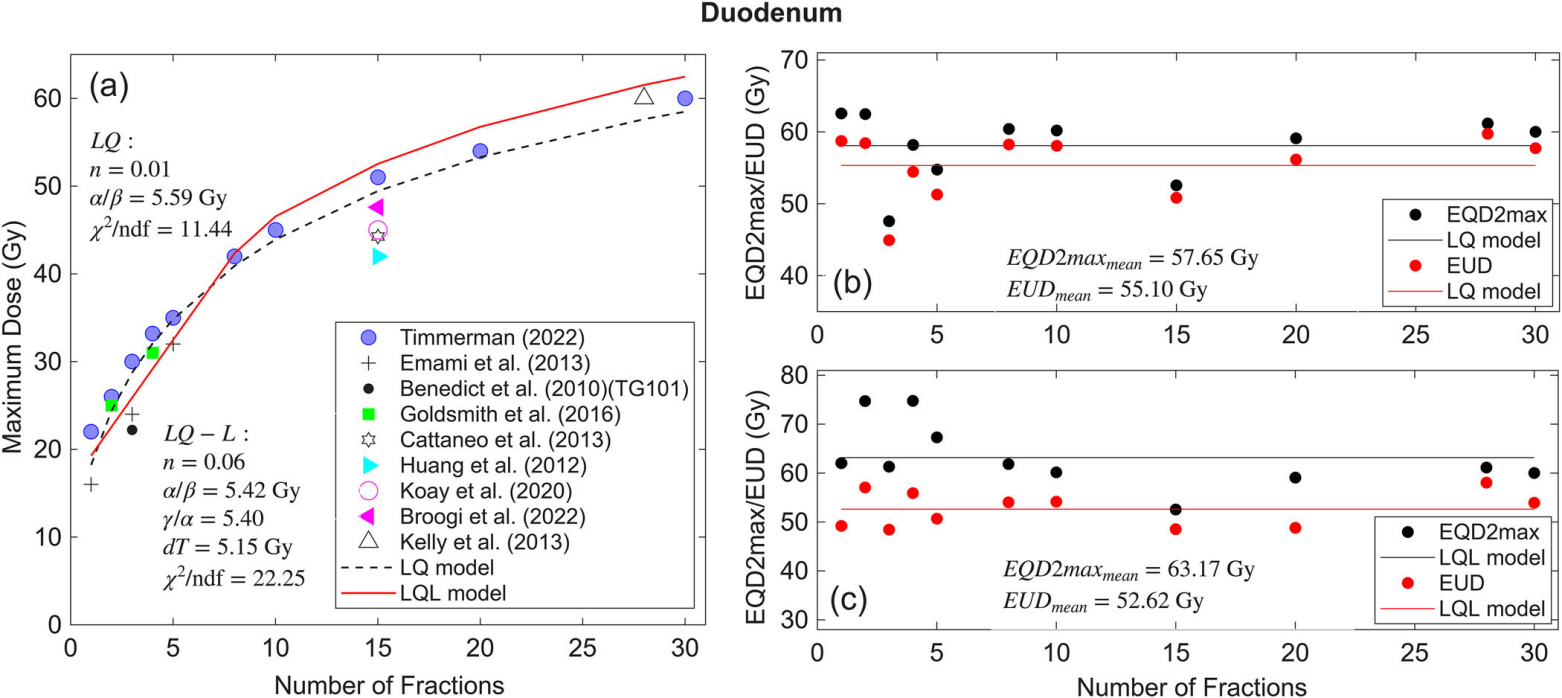
(a) Maximum dose vs number of fractions N, for duodenum calculated by LQ and LQ‐L models. (b) and (c) EQD2max/EUD vs the number of fractions calculated by LQ and LQL models. The Maximum dose and EQD2max correspond to the dose constraints with no more than 10% grade 3 toxicity. The reduced chi‐squared ($\chi^{2}/\mathrm{ndf}$) values are shown in plot (a), where ndf represents the number of degrees of freedom (number of data points minus the number of model parameters). The EQD2max and EUD values (black and red circles in Figure 1(b) and (c)) are summarized in Table 1.

The fitting results for duodenum (Figure 1) are plotted as maximum dose versus N (Figure 1(a)) and EQD2max/EUD versus N (Figure 1(b) and (c)). The EQD2max values are associated with the maximum dose. Maximum doses for duodenum, depicted in Figure 1(a), are sourced from the following Refs. 16, 19, 20, 21, 22, 23, 24, 25, 36 and the additional dose‐volume constraints used in the fitting are listed in the supplement (Table 1s).

In Figure 1(a), the solid red and dotted black curves indicate the LQ‐L and LQ models, respectively fitted to the published clinical data. Figure 1(b) and (c) shows EQD2max and EUD values for each fraction, along with mean EQD2max and mean EUD represented by black and red lines, respectively.

The LQ model parameters obtained from the fitting are α/β = 5.59 Gy (95% CI: 2.51, 8.68), and *n* = 0.01 (95% CI: −0.05, 0.07), with corresponding mean EUD and mean EQD2max values of 55.10 Gy (95% CI: 43.66, 66.65) and 57.65 Gy (95% CI: 52.63, 69.56), respectively.

For the LQ‐L model, parameters are α/β= 5.42 Gy (95% CI: −0.59, 11.45), γ/α = 5.40 (95% CI: 2.49, 8.31), $d_{t}$ = 5.15 Gy (95% CI: 2.94, 7.35), and *n* = 0.06 (95% CI: −0.05, 0.17), with corresponding mean EUD and mean EQD2max values of 52.62 Gy (95% CI: 33.81, 106.11) and 63.17 Gy (95% CI: 37.88, 111.58), respectively.

**TABLE 1:** Duodenum EQD2max and EUD values calculated using the LQ and LQ‐L models for various fractionation numbers (N).

N	1	2	3	4	5	8	10	15	20	28	30
EQD2max (Gy)	LQ: 62.58 LQL:62.04	LQ:62.48 LQL:74.71	LQ:47.56 LQL:63.17	LQ:58.09 LQL:74.76	LQ:54.74 LQL:67.28	LQ:60.42 LQL:61.83	LQ:60.18 LQL:60.14	LQ:52.57 LQL:52.63	LQ:59.10 LQL:59.0	LQ:61.16 LQL:61.15	LQ:60 LQL:60
EUD (Gy)	LQ:58.74 LQL:49.19	LQ:58.43 LQL:57.07	LQ:44.94 LQL:48.46	LQ:54.45 LQL:55.92	LQ:51.29 LQL:50.70	LQ:58.25 LQL:54.04	LQ:58.07 LQL:54.18	LQ:50.82 LQL:48.54	LQ:56.15 LQL:48.79	LQ:59.73 LQL:58.07	LQ:57.72 LQL:53.95

### 3.2 Stomach

**FIGURE 2:**
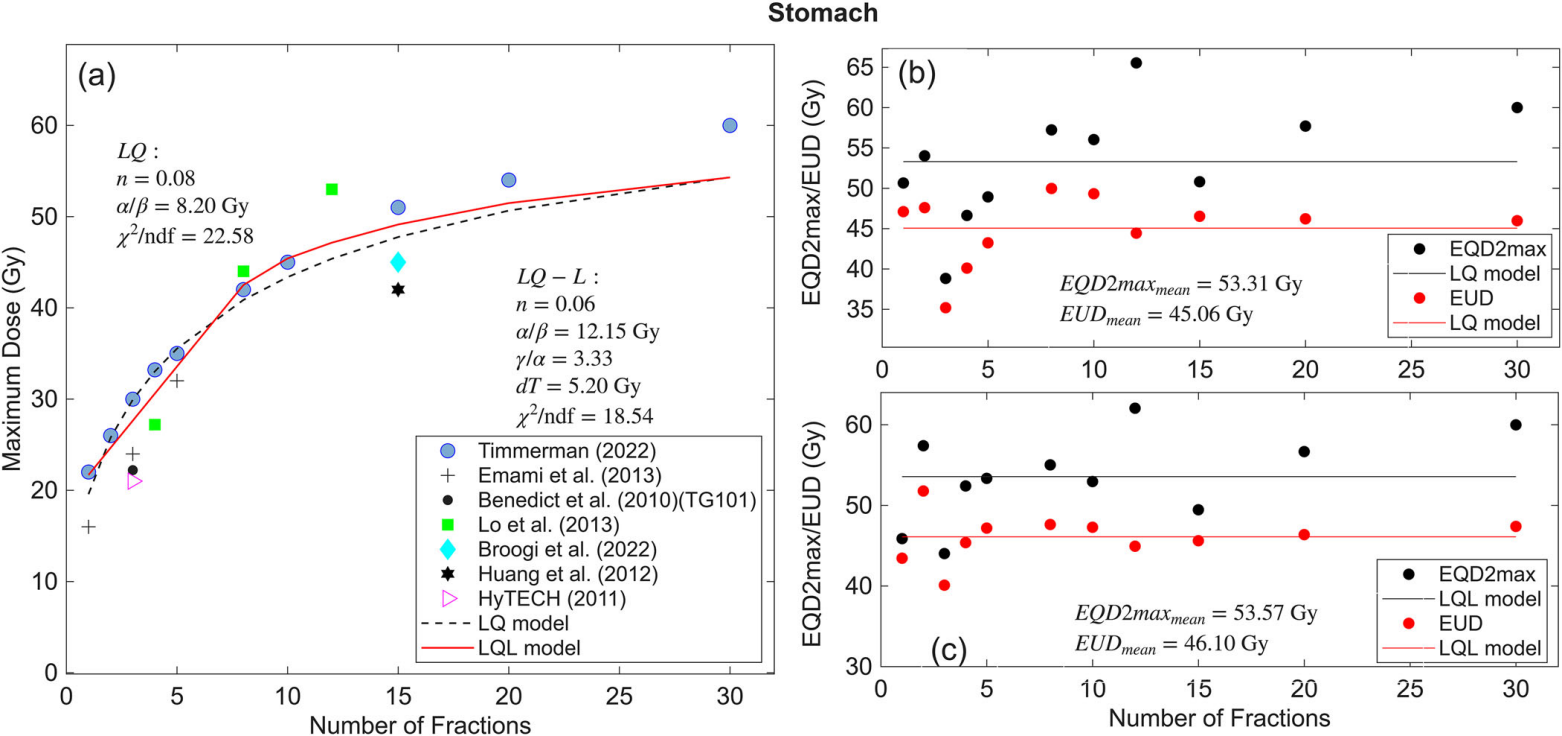
(a) Maximum dose vs number of fractions N, for stomach calculated by LQ and LQ‐L models. (b) and (c) EQD2max/EUD vs the number of fractions calculated by LQ and LQL models. The Maximum dose and EQD2max correspond to the dose constraints with no more than 10% grade 3 toxicity. The reduced chi‐squared ($\chi^{2}/\mathrm{ndf}$) values are shown in plot (a) where ndf represents the number of degrees of freedom (number of data points minus the number of model parameters). The EQD2max and EUD values (black and red circles in Figure 2(b) and (c)) are summarized in Table 2.

The stomach fitting results are depicted in Figure 2. The maximum doses shown in Figure 2(a) are obtained from the following Refs. 16, 17, 19, 20, 23, 25, 27 and the additional dose‐volume constraints used in the fitting are listed in the supplement Table 2s.

The solid red and dotted black curves in Figure 2(a) indicate the LQ‐L and LQ models, respectively fitted to the published clinical data. The black and red circles in Figure 2(b) and (c) represents the EQD2max and EUD in each fraction and the black and red lines show the mean EQD2max and mean EUD.

For the LQ model (dotted black curve), fitting yielded α/β = 8.20 Gy (95% CI: 4.45, 11.95), and *n* = 0.08 (95% CI: 0.01, 0.15), EUD = 45.06 Gy (95% CI: 40.1, 57.93), and EQD2max = 53.31 Gy (95% CI: 49.58, 61.37. For the LQ‐L model (solid red curve), α/β = 12.15 Gy (95% CI: −6.45, 30.76), γ/α = 3.33 (95% CI: 2.11, 4.56), $d_{t}$ = 5.20 Gy (95% CI: 3.68, 6.72) and *n *= 0.06 (95% CI: 0, 0.12), with corresponding EUD = 46.10 Gy (95% CI: 37.1, 74.75), and EQD2max = 53.57 Gy (95% CI: 44.96, 78.14).

**TABLE 2:** Stomach EQD2max and EUD values calculated using the LQ and LQ‐L models for various fractionation numbers (N).

N	1	2	3	4	5	8	10	12	15	20	30
EQD2max (Gy)	LQ:50.65 LQL:46.10	LQ: 53.31 LQL: 57.41	LQ: 38.83 LQL:44.03	LQ: 46.63 LQL:52.41	LQ: 48.93 LQL:53.35	LQ: 57.23 LQL:55.03	LQ: 56.04 LQL:52.96	LQ:65.55 LQL:62.05	LQ:50.81 LQL:49.47	LQ:57.70 LQL:56.68	LQ:60 LQL:60
EUD (Gy)	LQ: 47.09 LQL:43.44	LQ:47.60 LQL:51.78	LQ:35.21 LQL:40.11	LQ:40.10 LQL:45.39	LQ:43.23 LQL:47.18	LQ:49.98 LQL:47.62	LQ:49.31 LQL:47.29	LQ: 44.45 LQL:44.94	LQ: 46.52 LQL:45.61	LQ: 46.21 LQL:46.38	LQ: 45.98 LQL:47.39

### 3.3 Small bowel

**FIGURE 3:**
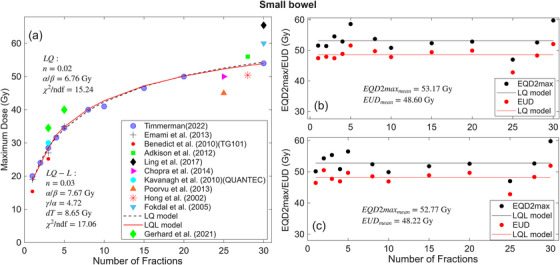
(a) Maximum dose versus number of fractions N, for small bowel calculated by LQ and LQ‐L models. (b) and (c) EQD2max/EUD vs number of fractions calculated by LQ and LQ‐L models. The Maximum dose and EQD2max correspond to the dose constraints with no more than 10% grade 3 toxicity. The reduced chi‐squared ($\chi^{2}/\mathrm{ndf}$) values are shown in plot (a), where ndf represents the number of degrees of freedom (number of data points minus the number of model parameters). The EQD2max and EUD values (black and red circles in Figure 3(b) and (c)) are summarized in Table 3.

In Figure 3(a) (maximum dose versus N) and Figure 3(b) and (c) (EQD2max/EUD versus N), the fitting results for small bowel are presented. The clinical data shown in Figure 3(a) are obtained from the following Refs. 16, 18, 19, 22, 28, 29, 30, 31, 32, 33, 34 and the additional dose‐volume constraints used in the fitting are listed in the supplement (Table 3s).

The solid red and dotted black curves in Figure 3(a) represent the LQ‐L and LQ models, respectively, fitted to the published clinical data. The black and red circles in Figure 3(b) and (c) represents the EQD2max and EUD in each fraction and the black and red lines show the mean EQD2max and mean EUD.

The results for the LQ model fitting have given an α/β = 6.76 Gy (95% CI: 4.89, 8.61), and *n* = 0.02 (95% CI: −0.02, 0.08), with the corresponding EUD and EQD2max values of 48.60 Gy (95% CI: 43.55, 54.61) and 53.17 Gy (95% CI: 50.83, 56.77), respectively. The LQ‐L fitting results are α/β = 7.67 Gy (95% CI: 3.80, 11.55), γ/α = 4.72 (95% CI: 3.13, 6.31), $d_{t}$ = 8.65 Gy (95% CI: 4.6, 12.68) and *n* = 0.03 (95% CI: −0.3, 0.09), EUD = 48.22 Gy (95% CI: 41.13, 66.02) and EQD2max = 52.77 Gy (95% CI: 48.23, 69.05).

**TABLE 3:** Small bowel EQD2max and EUD values calculated using the LQ and LQ‐L models for various fractionation numbers (N).

N	1	2	3	4	5	8	10	15	20	25	28	30
EQD2max (Gy)	LQ: 51.54 LQL:50.15	LQ: 51.41 LQL:54.29	LQ: 54.54 LQL:55.38	LQ: 52.90 LQL:50.86	LQ:58.60 LQL:56.49	LQ:53.70 LQL:52.40	LQ:50.83 LQL:49.89	LQ:52.34 LQL:51.78	LQ: 52.85 LQL:52.58	LQ: 46.96 LQL: 47.02	LQ:52.59 LQL:52.65	LQ: 59.78 LQL:59.78
EUD (Gy)	LQ: 47.46 LQL:46.44	LQ:47.91 LQL:50.52	LQ:47.43 LQL:47.73	LQ:48.84 LQL:46.95	LQ:51.56 LQL:49.68	LQ:49.75 LQL:48.53	LQ:47.80 LQL:46.92	LQ: 49.38 LQL:48.86	LQ: 49.92 LQL:49.67	LQ: 4279 LQL: 42.81	LQ: 48.30 LQL:48.33	LQ: 52.05 LQL:51.94

### 3.4 Summary of radiobiologic parameters

Table 4 shows a summary of the LQ‐L and LQ model parameters for the 3 OARs. The obtained reduced chi‐square ($\chi^{2}/ndf$) and EQD2max are also listed.

**TABLE 4:** The organ‐specific model parameters for three abdominal OARs using LQ‐L and LQ models.

		LQ‐L model							LQ model			
OAR	α/β (Gy)	γ/α	d_t_ (Gy)	*n*	EUD (Gy)	Χ^2^/*ndf*	EQD2 max (Gy)	α/β (Gy)	n	EUD (Gy)	$\chi^{2}/ndf$	EQD2 max (Gy)
Duodenum	5.42 CI (−0.59,11.45)	5.40 CI (2.49,8.31)	5.15 CI (2.94,7.35)	0.06 CI (−0.05,0.17)	52.62 CI: (33.81, 106.11)	5.22	63.17 CI: (37.88, 111.58)	5.59 CI:(2.51,8.68)	0.01 CI: (−0.05,0.07)	55.10 CI:(43.66,66.65)	8.15	57.65 CI:(52.63,69.56)
Stomach	12.15 CI: (−6.45,30.76	3.33 CI: (2.11,4.56)	5.20 CI (3.68,6.72)	0.06 CI (0,0.12)	46.10 CI(37.1,74.75)	2.79	53.57 CI(44.96, 78.14)	8.20 CI: (4.45,11.95)	0.08 CI: (0.01,0.15)	45.06 CI(40.1, 57.93)	5.70	53.31 CI(49.58,61.37)
Small bowel	7.67 CI: (3.80,11.55)	4.72 CI: (3.13,6.31)	8.65 CI (4.6,12.68)	0.03 CI (−0.3,0.09)	48.22 CI:(41.13, 66.02)	2.67	52.77 CI:(48.23,69.05)	6.76 CI: (4.89,8.61)	0.02 CI: (−0.02,0.08)	48.60 CI:(43.55,54.61)	2.55	53.17 CI: (50.83,56.77)

The EQD2max values are associated with the maximum doses that may lead to no more than 10% of grade 3 toxicity. $\chi^{2}/ndf$ is the reduced chi‐square with ndf being the number of degrees of freedom (number of data points minus the number of model parameters. The CI values are shown in brackets and represent the 95% confidence interval.

## 4 DISCUSSION

The approach employed in our current study to derive organ‐specific radiobiological parameters for three abdominal organs represents a novel methodology not previously utilized in similar investigations. For the first time, we extracted the EUD and radiobiological parameters *α/β*, *γ*, and volume effect parameter ‘*n*’ for each of the 3 OARs.

The *α/β* ratio of the stomach, as reported in the literature, typically falls within the range of 7–10 Gy.^39^ Our findings align closely with these established values, with our values yielding 8.20 Gy and 12.15 Gy for the LQ and LQ‐L models, respectively. Similarly, for the small bowel, our obtained *α/β* ratios of 6.76 and 7.67 Gy for the LQ and LQ‐L models, respectively, demonstrate consistency with the literature's reported range of 6–8.3 Gy.^39^

While the calculated volume effect parameter ‘*n*’ for the duodenum indicates a small value (0.01 and 0.05 for LQ and LQ‐L models, respectively), indicative of a serial organ, significant differences persist between the mean EQD2max and mean EUD. Specifically, as illustrated in Figure 1(b) and (c), these differences amount to 2.76 and 10.54 Gy for the LQ and LQ‐L models, respectively, emphasizing the importance of considering additional dose‐volume constraints beyond the maximum dose in determining toxicity. The same scenario was observed for the stomach and small bowel with the ‘*n*’ values of 0.08 and 0.06 for the LQ model (0.02 and 0.03 for the LQ‐L model). The differences between the mean EQD2max and mean EUD are 8.25 and 7.47 Gy for the stomach (Figure 2(b) and (c)) and 4.57 Gy and 4.55 Gy for small bowel in LQ and LQ‐L (Figure 3(b) and (c)) models, respectively.

It is significant to point out that in upper abdomen irradiation involving prolonged treatment time, acute toxicity may enhance late tissue effects, leading to what is known as consequential late effects. The deviation of *α*/*β* towards higher values than three suggests that more than one *α*/*β* component may be involved. The late toxicity rate includes a component that evolves from acute toxicity. This component, associated with the overall treatment time (OTT), has a larger *α*/*β* value. The combination of both acute and late effects may lead to an increase in the overall *α*/*β* ratio. Incorporating this effect in the EQD2 calculation would require adding OTT to the LQ/LQ‐L model, which would increase the number of parameters in the fitting process. While our current model does not include OTT in the EQD2 calculations, which represents a limitation of this study, we acknowledge that the clinical data used in the fitting may already reflect this effect, potentially explaining the observed increase in the *α*/*β* ratio for these OARs.

As we mentioned earlier, previous studies have employed the concept of mEUD as a predictor of toxicity, demonstrating its value in predicting adverse effects in cancer treatment planning.^13^, ^37^, ^38^, ^40^ For instance, Woody et al.^38^ studied the correlation of chest wall toxicity with different dose metrics, including mEUD, and found that mEUD improved the prediction of chest wall pain compared to other dose metrics. Similarly, Fleming et al.^12^ evaluated the predictive value of EUD for late bladder and rectal toxicity in patients with localized prostate cancer, showing that patients receiving an EUD above certain thresholds have a significantly higher risk of developing Grade 2+ late complications. We can further evaluate the correlation of EUD with toxicity in abdominal OARs using the individual DVH data alongside the EUD constraints and radiobiological parameters derived in this study.

A by‐product of the iso‐EUD fitting is the determination of the maximum EQD2, similar to the results reported in Ref. 8 where maximum physical dose constraints were fitted directly. This demonstrates that the iso‐EUD approach provides a more general framework for extracting radiobiological parameters for an OAR. As shown in Table 4, the $\chi^{2}/\mathrm{ndf}$ values for EUD fitting are generally lower for the LQ‐L model compared to the LQ model, except for the small bowel. Although the LQ‐L model offers a superior fit to the clinical dose–volume constraint data—particularly for the duodenum and stomach—the associated confidence intervals are notably wider than those from the simpler LQ model. This increased uncertainty likely arises from the larger number of fitted parameters in the LQ‐L model and the limited clinical data available for certain fractionation schemes. From a clinical perspective, the LQ‐L—derived EUD values remain valuable for treatment planning, as they better represent the underlying dose–response relationship across multiple fractionation regimens. However, the wider confidence intervals indicate that these estimates should be interpreted with caution. In practice, complementary metrics such as the LQ‐derived maximum EQD2 values, which are more stable and reproducible, can be used alongside EUD‐based constraints to guide plan evaluation and optimization.

According to the $\chi^{2}/\mathrm{ndf}$ values for the physical doses (see Figures 1, 2, and 3(a)), the LQ model predicts maximum doses better than the LQ‐L model for duodenum and small bowel. The $\chi^{2}/\mathrm{ndf}$ values are 22.25 (LQ‐L) and 11.44 (LQ) for duodenum, 18.54 (LQ‐L) and 22.58 (LQ) for stomach and 17.06 (LQ‐L) and 15.24 (LQ) for small bowel. These $\chi^{2}/\mathrm{ndf}$ values are comparable to ones obtained in Ref.,^8^ indicating the similar goodness of fittings.

The $\chi^{2}/\mathrm{ndf}$ values of LQ model are lower than those of the LQ‐L model for duodenum and small bowel. This observation can be attributed to the fact that the LQ model has simpler form and fewer parameters, which makes it more stable when fitting limited clinical data points at high doses. In contrast, the LQ‐L model introduces additional parameters to account for the linear behavior at high fractional doses. While this allows for a better overall fit to the full dose–volume data, it can also increase uncertainty in the predicted maximum doses, particularly when the number of high‐dose data points is limited. Therefore, for the available clinical dose range, the LQ model offers a more stable and reliable representation of maximum dose behavior.

In addition to highlighting the predictive power of EUD and the clinical utility of the results presented in this study, we would like to address some of its limitations. While EUD simplifies complex dose distributions into a single metric, it relies on averaging dose distributions and is highly dependent on the volume effect parameter ‘*n*’. For example, when ‘*n*’ is small, as in our study, the significance of hotspots increases for toxicity prediction, that is why we also provided the EQD2 to the small volume (0.03 cc), which is a useful clinical constraint for such organs. The Lyman model, though useful, relies on limited parameters and may fail to capture the biological complexities of tissues. Factors such as treatment timelines, chemotherapy, surgery, patient demographics, and smoking,^41^ which can significantly affect predictions, were not included in the current study. These limitations should be considered when interpreting results, emphasizing the need for future models to integrate additional parameters to improve clinical relevance.

A key consideration regarding the EUD model is that toxicity depends on both the volume effect and fractionation scheme, which are correlated. Ideally, the EUD model should incorporate both effects simultaneously. However, these factors are currently considered independently in our model, consistent with common practice in the literature.^13^, ^37^, ^38^ To obtain the mEUD, we first converted the dose matrix to EQD2 on a voxel‐by‐voxel basis to incorporate fractionation, then summed across volumes to determine the mEUD.

**FIGURE 4:**
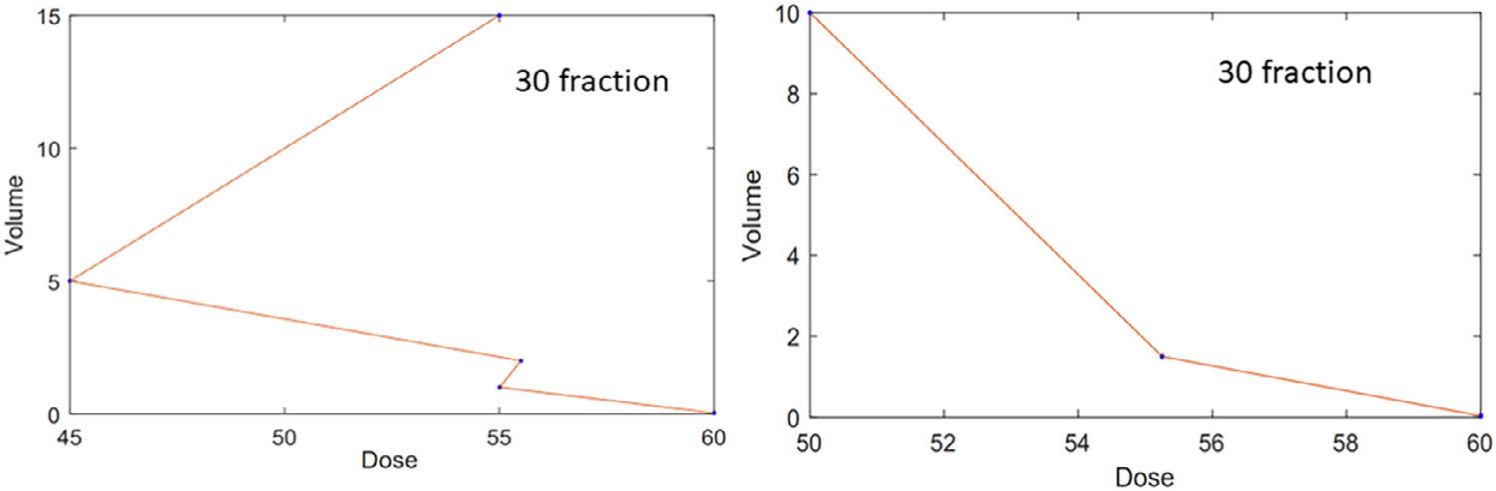
The left plot shows an example of DVH for duodenum with negative slopes. The right plot shows the modified DVH by connecting the middle points.

Due to limitations in clinical data meeting our selection criteria, certain challenges arose during the fitting process. In some fractions, dose‐volume constraints in the DVH exhibited negative slopes. To maintain these negative slopes, linear interpolation was conducted between the points (see Figure 4). While the ideal solution would involve polynomial fitting to the DVH points, the scarcity of data points precluded this approach. For some volumes in the DVH, various dose‐volume constraints from different references were identified. In such cases, the average values of the doses were utilized.

Furthermore, some of the data sources used in our study, such as the Emami and Timmerman tables, may raise concerns regarding their reliability. Although these tables have been widely used in clinics, as the author notes, most of their constraints are more educated guesses.^19^ Therefore, to assess the impact of Timmerman and Emami ‘s data on our results, we conducted a new fitting analysis for the duodenum, excluding these sources. Changes in both LQ and LQ‐L model results were minimal, indicating that excluding Timmerman and Emami ‘s data has only a small effect on the fitting outcomes.

Despite these limitations, the findings of this study provide a foundation for future clinical integration. The methodology and organ‐specific EUD constraints presented here have potential future clinical applications, particularly in biologically guided optimization and plan evaluation for abdominal radiotherapy. Integration of these parameters into inverse planning algorithms could help reduce toxicity risks and facilitate personalized treatment strategies across different fractionation schemes.

## 5 CONCLUSIONS

In this study, we applied a new approach to derive organ‐specific radiobiological parameters and EUD constraints from clinical data for three abdominal OARs, the duodenum, small bowel, and stomach. Our finding revealed *α/β* values that are consistent with those reported in previous studies, validating the robustness of the methodology. Additionally, the observed differences between EUD and EQD2max across all three OARs indicate the potential application of EUD constraints as valuable dose metrics for plan optimization and evaluation in radiation therapy.

By incorporating EUD constraints alongside traditional maximum doses, physicians and dosimetrists can enhance treatment planning accuracy and better predict radiation‐induced toxicity, ultimately improving patient outcomes. This novel approach opens avenues for further research and optimization in abdominal cancer treatment planning.

## AUTHOR CONTRIBUTIONS

**Samira Dabaghmanesh**: Investigation; formal analysis; original draft. **X. Allen Li**: Supervision; review & editing. **Eric S. Paulson**: Supervision; review & editing. **Beth Erickson**: Validation; review & editing. **William Hall**: Validation; review & editing. **An Tai**: Methodology; project formulation; supervision.

## CONFLICT OF INTEREST STATEMENT

The authors have no relevant conflicts of interest to disclose.

## Supporting information



Supporting Information

## Data Availability

All clinical data in this article can be found in Supplement
