# Supplementary material for: Organ‐specific equivalent uniform dose constraints and radiobiological parameters for radiation treatment planning of abdominal tumors
Source: J Appl Clin Med Phys. 2025 Dec 28;27(1):e70401. doi: 10.1002/acm2.70401 (PMC12744925; doi:10.1002/acm2.70401)
Supplement: Supplementary file 1 — Supporting Information [file ACM2-27-e70401-s001.docx]

# Supplement

Tables 1s–3s summarize published clinical data for the duodenum, stomach, and small bowel across various modalities, including CFRT, HFRT, and SBRT. These tables provide details such as the number of fractions, doses for specific volumes, the use of chemotherapy or surgery, and their respective references. When a reference specifies a type of chemotherapy, it is categorized as concurrent, adjuvant, or neoadjuvant; otherwise, it is noted as "chemotherapy." While most studies did not report overall treatment time, it can be estimated as approximately 1.4 times the number of fractions.

Table 1s. Duodenum

|  | N=1 | N=2 | N=3 | N=4 | N=5 | N=8 | N=10 | N=15 | N=20 | N=28 | N=30 | References |
| --- | --- | --- | --- | --- | --- | --- | --- | --- | --- | --- | --- | --- |
| D0.035 | 22 | 26 | 30 | 33.2 | 35 | 42 | 45 | 51 | 54 |  | 60 | \| Timmerman (1) \| \| --- \| |
|  | 16 |  | 24 |  | 32 |  |  |  |  |  |  | Emami (2) |
|  | 12.4 |  | 22.2 |  |  |  |  |  |  |  |  | Benedict et al. (3) |
|  |  | 25 |  | 31 |  |  |  |  |  |  |  | \| Goldsmith et al. (4) * \| \| --- \| |
|  |  |  |  |  |  |  |  | 44.3 |  |  |  | Cattaneo et al. (5) ** |
|  |  |  |  |  |  |  |  | 42 |  |  |  | Huang et al. (6) *** |
|  |  |  |  |  |  |  |  | 47.6 |  |  |  | Broggi et al. (7) ** |
|  |  |  |  |  |  |  |  | 45 |  |  |  | Koay et al. (8) * |
|  |  |  |  |  |  |  |  |  |  | 60 |  | Kelly et al. (9) *** |
|  |  |  |  |  |  |  |  |  |  |  |  |  |
| D0.5 cc |  |  |  |  | 30 |  |  |  |  |  |  | Gerhard et al. (10) |
|  |  |  |  |  |  |  |  | 45 |  |  |  | Liu et al. (11) **** |
|  |  |  |  |  | 33 |  |  |  |  |  |  | Diez et al. (12) |
|  |  |  |  |  |  |  |  |  |  |  |  |  |
| D1cc | 17 | 21.5 | 25.3 | 27 | 28 |  |  |  |  |  |  | \| Goldsmith et al. (4) * \| \| --- \| |
|  |  |  |  |  |  |  |  |  |  | 55 |  | Holyoake et al. (35) *** |
|  |  |  |  |  |  |  |  |  | 47.4 |  |  | Xia et al. (13) * |
|  |  |  | 21 |  |  |  |  |  |  |  |  | Gerhard et al. (10) |
|  |  |  |  |  | 25 |  |  |  |  |  |  | Gurka et al. (14) **** |
|  |  |  |  |  |  |  |  |  |  |  |  |  |
| D2cc |  |  |  |  |  |  |  |  |  |  | 55.5 | Nakamura et al. (15) * |
|  |  |  |  |  |  |  |  |  |  |  |  |  |
| D5cc | 17.4 | 20 | 22.5 | 25 | 26.5 | 31.2 | 33.9 | 39 | 42 |  | 45 | \| Timmerman (1) \| \| --- \| |
|  | 8.8 |  | 15 |  | 18 |  |  |  |  |  |  | Emami (2) |
|  | 11.2 |  | 16.5 |  |  |  |  |  |  |  |  | Benedict et al. (3) |
|  |  | 16.1 | 21 | 23.4 | 25.8 |  |  |  | 38.9 |  |  | Goldsmith16 (4) * |
|  |  |  |  |  | 18.3 |  |  |  |  |  |  | Gerhard et al. (10) |
|  |  |  |  |  |  |  |  |  |  |  |  |  |
| D10cc | 9 |  | 11.4 |  | 12.5 |  |  |  |  |  |  | Benedict et al. (3) (TG101) |
|  |  |  |  |  | 25 |  |  |  |  |  |  | Diez et al. (12) |
|  |  |  |  |  |  |  |  |  | 33.4 |  |  | Xia et al. (13) * |
|  |  |  |  |  |  |  |  |  |  |  |  |  |
| D15cc |  |  |  |  |  |  |  |  |  |  | 55 | Verma et al. (16) **** |
|  |  |  |  |  |  |  |  |  |  |  |  |  |
| D30cc |  | 12.5 | 15 | 17.5 | 20 |  |  |  |  |  |  | Goldsmith et al. (4) * |

* No chemoradiation or surgery, ** Concomitant chemoradiation, *** Concurrent, Neoadjuvant, Adjuvant chemo, **** Concurrent chemoradiation, ***** Chemoradiation, ****** Induction chemoradiation, ******* Concurrent chemoradiation and surgery.

Table 2s. Stomach

|  | N=1 | N=2 | N=3 | N=4 | N=5 | N=8 | N=10 | N=12 | N=15 | N=20 | N=30 | References |
| --- | --- | --- | --- | --- | --- | --- | --- | --- | --- | --- | --- | --- |
| D0.035 | 22 | 26 | 30 | 33.2 | 35 | 42 | 45 |  | 51 | 54 | 60 | \| Timmerman (1) \| \| --- \| |
|  | 16 |  | 24 |  | 32 |  |  |  |  |  |  | Emami (2) |
|  | 12.4 |  | 22.2 |  |  |  |  |  |  |  |  | Benedict et al. (3) |
|  |  |  |  | 27.1 |  | 44 |  | 53 |  |  |  | Lo et al. (17) |
|  |  |  |  |  |  |  |  |  | 42 |  |  | Huang et al. (6) *** |
|  |  |  |  |  |  |  |  |  | 45 |  |  | Broggi et al. (7) ***** |
|  |  |  | 21 |  |  |  |  |  |  |  |  | Choi et al. (18) ***** |
|  |  |  |  |  |  |  |  |  |  |  |  |  |
| D1cc |  |  |  |  |  |  |  |  |  | 51.2 |  | Xia et al. (13) * |
|  |  |  |  |  | 33 |  |  |  |  |  |  | Herman et al. (19) * |
|  |  |  |  |  |  |  |  |  |  |  |  |  |
| D2cc |  |  |  |  |  |  |  |  |  |  | 53.6 | Nakamura et al. (15) * |
|  |  |  |  |  |  |  |  |  | 46 |  |  |  |
|  |  |  |  |  |  |  |  |  | 44 |  |  | Broggi et al. (7) ** |
|  |  |  |  |  |  |  |  |  |  |  |  |  |
| D5cc | 17.4 | 20 | 22.5 | 25 | 26.5 | 31.2 |  |  |  |  |  | Timmerman (1) |
|  |  |  |  |  |  |  |  |  |  | 46.4 |  | \| Xia et al. (13) * \| \| --- \| |
|  |  |  |  |  | 30 |  |  |  |  |  |  | Chuong et al. (20) ****** |
|  |  |  | 21 |  |  |  |  |  |  |  |  | Grimm et al. (21) HyTEC2011 |
|  |  |  |  |  |  |  |  |  |  |  |  |  |
| D6cc |  |  |  |  |  |  |  | 30 |  |  |  | Shinoto et al. (22) **** |
|  |  |  |  |  |  |  |  |  |  |  |  |  |
| D10cc | 13 |  | 21 |  | 28 |  |  |  |  |  |  | \| Emami (2) \| \| --- \| \|  \| |
|  | 11.2 |  | 16.5 |  | 18 |  |  |  |  |  |  | Benedict et al. (3) |
|  |  |  |  |  |  |  |  |  |  | 42.7 |  | Xia et al. (13) * |
|  |  |  |  | 17.6 |  |  |  |  |  |  |  | Lo et al. (17) * |
|  |  |  | 22.5 |  |  |  |  |  |  |  |  | Gerhard et al. (10) |
|  |  |  |  |  | 26.5 |  |  |  |  |  |  | Gerhard et al. (10) |
|  |  |  |  |  | 25 |  |  |  |  |  |  | Hanna et al. (23) |
|  |  |  | 15 |  |  |  |  |  |  |  |  | Chang et al. (24) |
|  |  |  |  |  |  |  |  |  |  |  |  |  |
| D16cc |  |  |  |  |  |  |  |  |  |  | 50 | Nakamura et al. (15) * |
|  |  |  |  |  |  |  |  |  |  |  |  |  |
| D24cc |  |  |  |  |  |  |  | 20 |  |  |  | Shinoto et al. (22) ***** |
|  |  |  |  |  |  |  |  |  |  |  |  |  |
| D46cc |  |  |  |  |  |  |  |  |  |  | 40 | Nakamura et al. (15) * |
|  |  |  |  |  |  |  |  |  |  |  |  |  |
| D50cc |  |  |  |  |  |  | 33.9 |  | 39 | 42 | 45 | Timmerman (1) |
|  |  |  |  |  |  |  |  |  |  |  |  |  |
| D94cc |  |  |  |  |  |  |  |  |  |  | 30 | Nakamura et al. (15) * |
|  |  |  |  |  |  |  |  |  |  |  |  |  |
| D102cc |  |  |  |  |  |  |  | 10 |  |  |  | Shinoto et al. (22) **** |
| D119cc |  |  |  |  |  |  |  |  |  |  | 20 | Nakamura et al. (15) * |

* No chemoradiation or surgery, ** Concomitant chemoradiation, *** Concurrent, Neoadjuvant, Adjuvant chemo, **** Concurrent chemoradiation, ***** Chemoradiation, ****** Induction chemoradiation, ******* Concurrent chemoradiation and surgery.

Table 3s. Small bowel

|  | N=1 | N=2 | N=3 | N=4 | N=5 | N=8 | N=10 | N=15 | N=20 | N=25 | N=28 | N=30 | References |
| --- | --- | --- | --- | --- | --- | --- | --- | --- | --- | --- | --- | --- | --- |
| D0.035cc | 20 | 24 | 28.5 | 31.6 | 34.5 | 40 | 41 | 46.5 | 50 |  |  | 54 | \| Timmerman (1) \| \| --- \| |
|  | 19 |  | 27 |  | 35 |  |  |  |  |  |  |  | Emami (2) |
|  | 15.4 |  | 25.2 |  |  |  |  |  |  |  |  |  | Benedict et al. (3) |
|  |  |  |  |  |  |  |  |  |  |  | 56 |  | Adkison et al. (25) * |
|  |  |  |  |  |  |  |  |  |  |  |  | 65.5 | Ling et al. (26) **** |
|  |  |  |  |  |  |  |  |  |  | 50 |  |  | Chopra et al. (27) ******* |
|  |  |  |  |  |  |  |  |  |  | 45 |  |  | Poorvu et al. (28) **** |
|  |  |  |  |  |  |  |  |  |  | 45 |  |  | \| Kavanagh et al. (29) (QUANTEC) \| \| --- \| |
|  |  |  | 30 |  |  |  |  |  |  |  |  |  | Kavanagh et al. (29) (QUANTEC) |
|  |  |  |  |  |  |  |  |  |  |  | 50.4 |  | Hong et al. (30) * |
|  |  |  |  |  | 40 |  |  |  |  |  |  |  | Gerhard et al. (10) |
|  |  |  | 34.5 |  |  |  |  |  |  |  |  |  | Gerhard et al. (10) |
|  |  |  |  |  |  |  |  |  |  |  |  | 60 | Fokdal et al. (34) * |
|  |  |  |  |  |  |  |  |  |  |  |  |  |  |
| D0.5cc |  |  |  |  |  |  |  |  |  |  |  |  | Gerhard et al. (10) |
|  |  |  |  |  |  |  |  |  |  |  |  |  |  |
| D1cc |  |  | 21 |  |  |  |  |  |  |  |  |  | Gerhard et al. (10) |
|  |  |  | 30 |  |  |  |  |  |  |  |  |  | Gerhard et al. (10) |
|  |  |  |  |  | 35 |  |  |  |  |  |  |  | Chuong et al. (20) ****** |
|  |  |  |  |  |  |  |  |  |  |  |  | 60 | Poorvu et al. (28) **** |
|  |  |  |  |  |  |  |  |  |  |  |  |  |  |
| D2cc |  |  |  |  |  |  |  |  |  |  |  | 53.7 | Nakamura et al. (15) * |
|  |  |  |  |  |  |  |  |  |  |  |  |  |  |
| D5cc | 9.8 |  | 16.2 |  | 19.5 |  |  |  |  |  |  |  | Emami (2) |
|  | 11.9 |  | 17.7 |  |  |  |  |  |  |  |  |  | Benedict et al (3) |
|  |  |  |  |  | 30 |  |  |  |  |  |  |  | Chuong et al. (20) ****** |
|  |  |  |  |  |  |  |  |  |  |  |  |  |  |
| D5.3cc |  |  |  |  |  |  |  |  |  |  |  | 55 | Poorvu et al. (28) **** |
|  |  |  |  |  |  |  |  |  |  |  |  |  |  |
| D10cc |  |  |  |  |  |  |  |  |  |  |  | 50 | Nakamura et al. (15) * |
|  |  |  |  |  | 25 |  |  |  |  |  |  |  | Diez et al. (12) |
|  |  |  |  |  |  |  |  |  |  |  |  |  |  |
| D20cc |  |  |  |  | 28.5 |  |  |  |  |  |  |  | Gerhard et al. (10) |
|  |  |  |  |  |  |  |  |  |  |  |  |  |  |
| D30cc | 17.6 | 19.2 | 20.7 | 22.4 | 24 | 28.8 |  |  |  |  |  |  | Timmerman (1) |
|  | 12.5 |  |  |  |  |  |  |  |  |  |  |  | Kavanagh et al. (29) (QUANTEC) |
|  |  |  | 17.4 |  |  |  |  |  |  |  |  |  | Gerhard et al. (10) |
|  |  |  |  |  | 20 |  |  |  |  |  |  |  | Gerhard et al. (10) |
|  |  |  |  |  |  |  |  |  |  |  |  |  |  |
| D75cc |  |  |  |  |  |  |  |  |  | 40 |  |  | Chopra et al. (31) **** |
|  |  |  |  |  |  |  |  |  |  |  |  |  |  |
| D120cc |  |  |  |  |  |  | 33.9 | 39 | 42 |  |  | 45 | Timmerman (1) |
|  |  |  |  |  |  |  |  |  |  |  |  |  |  |
| D150cc |  |  |  |  |  |  |  |  |  |  |  | 15 | Baglan et al. (32) **** |
|  |  |  |  |  |  |  |  |  |  |  |  |  |  |
| D190cc |  |  |  |  |  |  |  |  |  | 30 |  |  | Chopra et al. (33) **** |
|  |  |  |  |  |  |  |  |  |  |  |  |  |  |
| D275cc |  |  |  |  |  |  |  |  |  | 15 |  |  | Chopra et al. (27) ******* |
|  |  |  |  |  |  |  |  |  |  |  |  |  |  |
| D372.4cc |  |  |  |  |  |  |  |  |  |  | 30 |  | Adkison et al. (25) * |

* No chemoradiation or surgery, ** Concomitant chemoradiation, *** Concurrent, Neoadjuvant, Adjuvant chemo, **** Concurrent chemoradiation, ***** Chemoradiation, ****** Induction chemoradiation, ******* Concurrent chemoradiation and surgery.

# References

1. Timmerman R. A story of hypofractionation and the table on the wall. *Int J Radiat Oncol Biol Phys.* 2022; 112(1):4-21. doi: 10.1016/j.ijrobp.2021.09.027

2. Emami B. Tolerance of normal tissue to therapeutic radiation. *Reports Radither Oncol.* 2013 1:35-48. doi: 10.1016/0360-3016(91)90171-y

3. Benedict SH, Yenice KM, Followill D, Galvin JM, Hinson W, Kavanagh B, et al. Stereotactic body radiation therapy: The report of AAPM Task group 101. *Medical Physics.* 2010; 37(8):4078-101. doi: 10.1118/1.3438081

4. Goldsmith C, Price P, Cross T, Loughlin S, Cowley I, Plowman N. Dose-volume histogram analysis of stereotactic body radiotherapy treatment of pancreatic cancer: a focus on duodenal dose constraints. *Semin Radiat Oncol.* 2016; 26:149-56. doi: 10.1016/j.semradonc.2015.12.002.

5. Cattaneo GM, Passoni P, Longobardi B, Slim N, Reni M, Cereda S, et al. Dosimetric and clinical predictors of toxicity following combined chemothrapy and moderately hypofractionated rotational radiotherapy of locally advanced pancreatic adenocarcinoma. *Radiotherapy and Oncology.* 2013; 108(1):66-71. doi: 10.1016/j.radonc.2013.05.011

6. Huang J, Robertson JM, Ye H, Margolis J, Nadeau L, Yan D. Dose-volume analysis of predictors for gastrointestinal toxicity after concurrent full-dose gemcitabine and radiotherapy for locally advanced pancreatic adenocarcinoma. *Int J Radiat Oncol Biol Phys.* 2012; 83(4):1120-5. doi: 10.1016/j.ijrobp.2011.09.022

7. Broggi S, Passoni P, Tiberio P, Cicchetti A, Cattaneo GM, Longobardi B, et al. Stomach and duodenum dose-volume constraints for locally advanced pancreatic cancer patients treated in 15 fractions in combination with chemotherapy. *Front. Oncol.* 2023; 12:983-984. doi: 10.3389/fonc.2022.983984. eCollection 2022

8. Koay EJ, Hanania AN, Hall WA, Taniguchi CM, Rebueno N, Myrehaug S, et al. Dose-escalated Radiation Therapy for Pancreatic Cancer: A simultaneous Integrated Boost Approach. *Pract Radiat Oncol.* 2020; 10(6):495-507. doi: 10.1016/j.prro.2020.01.012

9. Kelly P, Das P, Pinnix CC, Beddar S, Briere T, Pham M, et al. Duodenal toxicity after fractionated chemoradiation for unresectable pancreatic cancer. *Int. J. Radiat. Oncol. Biol. Phys.* 2013; 85:143-9. doi: 10.1016/j.ijrobp.2012.09.035

10. Gerhard SG, Palma DA, Arifin AJ, Louie AV, Li GJ, Al-Shafa F, et al. Organ at risk Dose Constraints in SABR: a systematic Review of Active Clinical Trials. *Practical Radiation Oncology.* 2021; 11:355-365. doi: 10.1016/j.prro.2021.03.005

11. Liu X, Ren G, Li L, Xia T. Predictive dosimetric parameters for gastrointestinal toxicity with hypofractioned radiotherapy in pancreatic adenocarcinoma. *Onco Targets Ther.* 2016; 9:2489-94. doi: 10.2147/OTT.S102035.

12. Diez P, Hanna GG, Aitken KL, van As N, Carver A, Colaco RJ, et al. UK 2022 Consensus on Normal Tissue Dose-Volume Constraints for Oligometastatic, Primary Lung and Hepatocellular Carcinoma Stereotactic Ablative Radiotherapy. *Clin. Oncol.* 2022; 34:288-300. doi: 10.1016/j.clon.2022.02.010

13. Xia T, Chang D, Wang Y, Li J, Wu W, Zhu F, et al. Dose escalation to target volumes of helical tomotherapy for pancreatic cancer in the phase 1-2 clinical trial. *Int J Radiat Oncol Biol Phys.* 2013; 87:303.

14. Gurka MK, Collins SP, Slack R, Tse G, Charabaty A, Ley L, et al. Stereotactic body radiation therapy with concurrent full-dose gemcitabine for locally advanced pancreatic cancer: a pilot trial demonstrating safety. *Radiat Oncol.* 2013; 8:44. doi: 10.1186/1748-717X-8-44. PMID: 23452509

15. Nakamura A, Shibuya K, Matsuo Y, Nakamura M, Shiinoki T, Mizowaki T, et al. Analysis of dosimetric parameters associated with acute gastrointestinal toxicity and upper gastrointestinal bleeding in locally advanced pancreatic cancer patients treated with gemcitabine-based concurrent chmoradiotehrapy. *Int J radiat Oncol Biol Phys.* 2012; 84(2):369-75. doi: 10.1016/j.ijrobp.2011.12.026

16. Verma J, Sulman EP, Jhingran A, Tucker SL, Rauch GM, Eifel PJ, et al. Dosimetric predictors of duodenal toxicity after intensity modulated radiation therapy for treatment of the paraaortic nodes in gyneocologic cancer. *Int J Radiat Oncol. Biol. Phys.* 2014; 88(2):357-62. doi: 10.1016/j.ijrobp.2013.09.053

17. Lo SS, Sahgal A, Chang EL, Mayr NA, Teh BS, Huang Z, et al. Serious complication associated with stereotactic ablative radiotherapy and strategies to mitigate the risk. *Clin. Oncol. (R Coll Radiol).* 2013; 25(6):378-87. doi: 10.1016/j.ijrobp.2012.09.035

18. Choi BO, Choi IB, Jang HS, Kang YN, Jang JS, Bae SH, et al. Stereotactic body radiation therapy with or without transarterial chemoembolization for patients with primary hepatocellular carcinoma: preliminary analysis. BMC Cancer. (2008) 8(351). doi:10.1186/1471-2407-8-351

19. Herman JM, Chang DT, Goodman KA, Dholakia AS, Raman SP, Hacker-Prietz A, et al. Phase 2 multi-institutional trial evaluating gemcitabine and stereotactic body radiotherapy for patients with locally advanced unresectable pancreatic adenocarcinoma. *Cancer.* 2015; 121(7):1128-1137. doi: 10.1002/cncr.29161

20. Chuong MD, Springett GM, Freilich JM, Park CK, Weber JM, Mellon EA, et al. Stereotactic body radiation therapy for locally advanced and borderline resectable pancreatic cancer is effective and well tolerated. *Int J Radiat Oncol Biol Phys.* 2013; 86(3):516-522. doi: 10.1016/j.ijrobp.2013.02.022

21. Grimm J, LaCouture T, Croce R, Yeo I, Zhu Y, Xue J. Dose tolerance limits and dose volume histogram evaluation for stereotactic body radiotherapy. *J Appl Clin Med Phys.* 2011; 12(2):33-68. doi: 10.1120/jacmp.v12i2.3368

22. Shinoto M, Shioyama Y, Matsunobu A, Okamoto K, Suefuji H, Toyama S, et al. Dosimetric analysis of upper gastrointestinal ulcer after carbon-ion radiotherapy for pancreatic cancer. *Radiotherapy and Oncology,* 2016; 120(1):140-144. doi: 10.1016/j.radonc.2016.04.040

23. Hanna GG, Murray L, Patel R, Jain S, Aitken KL, Franks KN, et al. UK Consensus on Normal Tissue Dose Constraints for Stereotactic Radiotherapy. *Clinical Oncology.* 2018; 30(1):5-14. doi: 10.1016/j.clon.2017.09.007

24. Chang BK, Timmerman RD, Stereotactic body radiation therapy: a comprehensive review. *Am J Clin Oncol.* 2007; 30(6):637-44. doi: 10.1097/COC.0b013e3180ca7cb1

25. Adkison JB, McHaffie DR, Bentzen SM, Patel RR, Khuntia D, Petereit DG, et al. Phase I trial of pelvic nodal dose escalation with hypofractionated IMRT for high-risk prostate cancer. *Int J Radiat Oncol Biol Phys.* 2012; Jan 1;82(1):184-90. doi: 10.1016/j.ijrobp.2010.09.018.

26. Ling A, Furhang E, Ryemon SN, Ennis RD. Late small toxicity after aggressive abdominpelvic intensiti modulated radiation therapy. *Adv. Radiat. Oncol.* 2017; 2(4):615-623. doi: 10.1016/j.adro.2017.09.005. eCollection 2017 Oct-Dec.

27. Chopra S, Dora T, Chinnachamy AN, Thomas B, Kannan S, Engineer R, et al. Predictors of grade 3 or higher late bowel toxicity in patients undergoing pelvic radiation for cervical cancer: results from a prospective study. *Int J radiat Oncol Biol Phys.* 2014; 88(3):630-635. doi: 10.1016/j.ijrobp.2013.11.214

28. Poorvu PD, Sadow CA, Townamchai K, Damato AL, Viswanathan AN. Duodenal and other gastrointestinal toxicity in cervical and endometrial cancer treated with extended-field intensity modulated radiation therapy to paraaortic lymph nodes. *Int J Radiat Oncol Biol Phys.* 2013; 85:1262-68. doi: 10.1016/j.ijrobp.2012.10.004.

29. Kavanagh BD, Pan CC, Dawson LA, Das SK, Li XA, Ten Haken RK, et al. Radiation dose-volume effects in the stomach and small bowel*. Int J Radiat Oncol Biol Phys.* 2010; 76:101-7. doi: 10.1016/j.ijrobp.2009.05.071

30. Hong JH, Tsai CS, Lai CH, Chang TC, Wang CC, Lee SP, et al. Postoperative low-pelvic irradiation for stage I-IIA cervical cancer patients with risk factors other than pelvic lymph node metastasis. *Int J Radiat Oncol Biol Phys.* 2002; 53(5):1284-90. doi: 10.1016/s0360-3016(02)02831-6

31. Chopra S, Gupta S, Kannan S, Dora T, Engineer R, Mangaj A, et al. Late Toxicity After Adjuvant Conventional Radiation Versus Image-Guided Intensity-Modulated Radiotherapy for Cervical Cancer (PARCER): A Randomized Controlled Trial. *J Clin Oncol.* 2021; 39(33):3682-3692. doi: 10.1200/JCO.20.02530

32. Baglan KL, Frazier RC, Yan D, Huang RR, Martinez AA, Robertson JM. et al. The dose-volume relationship of acute small bowel toxicity from concurrent 5-FU-based chemotherapy and radiation therapy for rectal cancer. *In J Radiat Oncol Biol Phys.* 2002; 52:176-183. doi: 10.1016/s0360-3016(01)01820-x

33. Chopra S, Krishnatry R, Dora T, Kannan S, Thomas B, Sonawone S, et al. Predictors of late bowel toxicity using three different methods of contouring in patients undergoing post-operative radiation for cervical cancer. *Br J Radiol.* 2015; 88(1055):20150054. doi: 10.1259/bjr.20150054

34. Fokdal L, Honoré H, Høyer M, von der Maase H. Dose-volume histograms associated to lung-term colorectal functions in patients receiving pelvic radiotherapy. *Radiother. Oncol.* 2005; 74(2):203-210. doi: 10.1016/j.radonc.2004.11.001

35. Holyoake DLP, Aznar M, Mukherjee S, Partridge M, Hawkins MA. Modeling duodenum radiotherapy toxicity using cohort dose-volume-histogram data. *Radiother. Oncol.* 2017; 123(3):431-437. doi: 10.1016/j.radonc.2017.04.024
